# Design of Customized Mouthguards with Superior Protection Using Digital-Based Technologies and Impact Tests

**DOI:** 10.1186/s40798-024-00728-2

**Published:** 2024-05-31

**Authors:** Naser Nasrollahzadeh, Dominique P. Pioletti, Martin Broome

**Affiliations:** 1 Division of Oral & Maxillofacial surgery, Lausanne University Hospital (CHUV) and Lausanne University, Rue du Bugnon 44, Lausanne, 1011 Switzerland; 2 Laboratory of Biomechanical Orthopedics, Institute of Mechanical Engineering, EPFL, Lausanne, Switzerland

**Keywords:** Customized Mouthguard, Finite Element Analysis, 3D Printing, Impact test, Dental Protection, Load Distribution, Energy Absorption

## Abstract

**Background:**

In contact sports, an impact on the jaw can generate destructive stress on the tooth-bone system. Mouthguards can be beneficial in reducing the injury risk by changing the dynamics of the trauma. The material properties of mouthguards and their geometrical/structural attributes influence their protective performance. Custom-made mouthguards are the gold standard, and different configurations have been proposed to improve their protection and comfort. However, the effects of different design variables on the performance of customized mouthguards are not well understood.

**Results:**

Herein, we developed a reliable finite element model to analyze contributing factors to the design of custom-made mouthguards. Accordingly, we evaluated the isolated and combined effect of layers’ stiffness, thickness, and space inclusion on the protective capability of customized mouthguards. Our simulations revealed that a harder frontal region could distribute load and absorb impact energy through bending if optimally combined with a space inclusion. Moreover, a softer layer could enlarge the time of impact and absorb its energy by compression. We also showed that mouthguards present similar protection with either permanently bonded or mechanically interlocked components. We 3D-printed different mouthguards with commercial resins and performed impact tests to experimentally validate our simulation findings. The impact tests on the fabricated mouthguards used in this work revealed that significantly higher dental protection could be achieved with 3D-printed configurations than conventionally fabricated customized mouthguards. In particular, the strain on the impacted incisor was attenuated around 50% more with a 3D-printed mouthguard incorporating a hard insert and space in the frontal region than a conventional Playsafe® Heavypro mouthguard.

**Conclusions:**

The protective performance of a mouthguard could be maximized by optimizing its structural and material properties to reduce the risk of sport-related dental injuries. Combining finite element simulations, additive manufacturing, and impact tests provides an efficient workflow for developing functional mouthguards with higher protectiveness and athlete comfort. We envision the future with 3d-printed custom-mouthguards presenting distinct attributes in different regions that are personalized by the user based on the sport and associated harshness of the impact incidences.

## Background

Athletes involved in various sporting activities may be prone to orofacial injuries in the case of a traumatic jaw impact. They can wear a mouthguard to reduce dental damage upon oral impact. However, there is a widespread reluctance among them to use a mouthguard despite an awareness of its protective advantages. Their bulky nature and the resulting perceived discomfort are often mentioned. Factors contributing to athletes’ comfort include mouthguard retention, appearance, occlusal/labial feel, and ease of breathing and speaking, which can influence athletes’ field performance [1, 2].

Available mouthguards, including off-the-shelf and custom-made ones, require compromises in cost, comfort, protection level, and lead time. Non-customized mouthguards, such as off-the-shelf or boil-and-bite varieties, are affordable and readily available but are uncomfortable and do not provide optimal protection [2–4]. Custom-made mouthguards are conventionally fabricated based on lamination of thermoplastic sheets (e.g., Ethylene-vinyl acetate-EVA) over the 3D impression model of a wearer’s teeth through a costly and time-consuming process. While being the current gold standard, maintaining uniform and accurate dimensions of the final customized mouthguards during the thermoforming process is still problematic [4, 5]. Over the last few years, digital manufacturing technologies such as 3D printing have increased the available possibilities for cost-effective and easy fabrication of customized mouthguards with required geometrical accuracy [6–8]. However, 3D-printed mouthguards have not been clinically evaluated for reducing the risk of dental trauma in sports. The limited availability of biocompatible materials to 3D-print functional mouthguards and the immature manufacturing workflow is often mentioned as the main barriers to the integration of these devices in the sports industry [4, 9, 10].

Several studies have compared the protective performance of conventionally fabricated custom-made mouthguards having different design configurations. Previous studies [11–14] have consistently shown that thicker mouthguards are more protective than thinner ones. A mouthguard thickness of 4 mm is generally considered a compromised value for protection and comfort. Likewise, space inclusion within raw EVA layers was reported as a contributing factor in reducing the transmitted force during an impact [15]. However, the isolated effect of space inclusion on the protectiveness of customized mouthguards has never been clinically evaluated. While some studies showed a beneficial effect of a sandwiched hard insert between soft layers for protection against high-impact energies [11], some others reported detrimental or non-significant effects of this reinforced lamination design [13, 16]. Additionally, the combination of a hard insert and an intentional space in the frontal region was suggested to reduce transmitted stress to the teeth following a direct impact [13, 17, 18]. It is worth mentioning that with the conventional thermoforming method, it is impossible to assess the effects of an isolated space or hard insert incorporation without changing the total thickness of the customized mouthguard. For instance, the effect of space inclusion is always combined with the projected frontal region thickness [11] due to thermoforming of materials on offset frontal teeth [19].

Due to ethical reasons and the high risk of injuries, the implementation of jaw impact tests on volunteers is impossible. Alternative methods should be employed to evaluate mouthguards’ protective performance. Digital-based modeling and finite element (FE) impact simulations provide powerful tools to evaluate the effect of different design variables on mouthguards protective performance. In particular, previous studies employed FE analysis to compare custom-made and off-the-shelf mouthguards for dental trauma prevention [14], to evaluate dentoalveolar model for impact testing of mouthguards [20], and to predict the effect of mouthguard thickness on its protective performance [12]. Indeed, the complexity of the FE model and the type of impact loading can significantly influence the results of the simulation and its prediction capability. In this regard, the performed FE simulations are limited to static impact analysis [14], 2D modeling of dentoalveolar cross-Sect. [20] and too simplified 3D dental models without inclusion of neighbouring hard or soft tissues [12]. Rationally, a fairly simple 3D model of teeth-bone system protected with mouthguard and a reliable dynamic impact simulation can highlight the role of potential contributing factors such as hard insert or space inclusion in the design. With such a model in hand, the level of transmitted stress/strain on impacted teeth could be analysed. Undeniably, measuring the affected teeth response upon an impact is the most reliable metric to estimate mouthguards functionality for dental trauma prevention.

In this study, we evaluated the role of different design variables in the shock absorbing capability of mouthguard using numerical simulations and experimental tests. The study was structured in 3 successive steps. First, a 3D human jaw finite element model was developed and different impacts were simulated on bare and protected teeth by changing the configuration of mouthguards. Having analysed the effect of thickness, material properties and space inclusion on mouthguards performance, we found that maximal protection could be obtained in a composite mouthguard by optimizing its structural design and material properties. Second, we fabricated representative custom-made mouthguards using the 3D-printing method to experimentally evaluate their performance and confront them with our simulation findings. Third, the protective capabilities of the 3D-printed and conventional customized mouthguards were compared by impact tests.

## Methods

### Anatomical Model Development for Impact Simulation

**Fig. 1:**
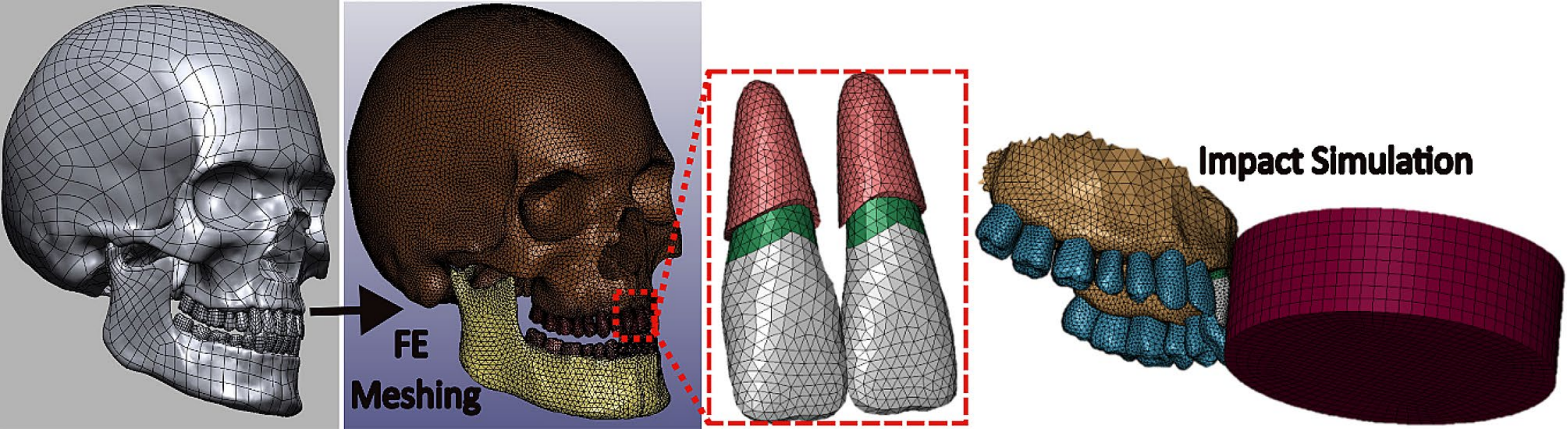
Anatomical human skull, jaw, teeth, and periodontal ligament (PDL) finite element models for direct puck impact simulation. Incisors comprise dentin (green) and enamel (white) parts. The PDL (pink) covers the root of the incisors and connects them to the maxilla bone

To study in silico the impact on the jaw with and without the presence of a mouthguard, an anatomical human head FE model was developed, including the skull, teeth, and periodontal ligament (PDL), as shown in Fig. 1. To closely mimic the anatomical shape of the head components, the model was meshed by tetrahedral elements with good quality (e.g., 99.9% of maxilla, teeth and PDL elements having aspect ratio less than 3 and 94% angle range between 30- to105 degree). Knowing that most dental traumas affect incisors [11, 20, 21], we only considered PDL for incisors to estimate the impact-induced stress on them more reliably. Indeed, by excluding the PDL of other teeth, we reduced the unnecessary complexity of the model, which can otherwise impose high computational costs. The PDL component was tied to incisors’ root and maxilla bone over the internal and external interfaces, respectively.

Mechanical behavior of bone, PDL, and teeth (enamel and dentin) were considered isotropic and linearly elastic, and their material properties were assigned according to the literature [12, 14, 22]. Table 1 shows the indicative parameters of the developed FE model used during impact simulations. The simulations were performed on the extracted maxilla part to reduce computational time since we were interested in the incisor’s stress/strain field following an impact. The maxilla part was fixed from the back side in all degrees of freedom, and the impacts were simulated with LS-DYNA explicit solver (Ansys, Pennsylvania). A surface-to-surface contact [23] was defined between the puck and the impacted components. The puck was considered a viscoelastic material as reported in other studies for dynamic impact simulations [24]. The initial speed of the puck was 3 m/s for all simulations, and the resulting impact energy was 0.75 J.

**Table 1:** Material models and indicative mechanical properties of different parts in the developed FE model

Part	Material Model	Indicative property	Part	Material Model	Indicative property
PDL	Elastic-Mat001	E = 50 MPa (14, 22)	**Puck**	Viscoelastic-Mat006	${G}_{0}=414 \& {G}_{\infty }=26.9 \text{M}\text{P}\text{a} \left(24\right)$
Enamel	Elastic-Mat001	E = 80 GPa (12, 22)	**EVA**	Sim. Rubber-Mat181	E = 18 MPa, Fig. 3-a
Dentin	Elastic-Mat001	E = 18 GPa (12, 22)	**SBS**	Plasticity-Mat124	E = 1.8 GPa, Fig. 3-b
Bone	Elastic-Mat001	E = 13.7 GPa (14, 22)	**PC**	Elastic-Mat001	E = 2.4 or 3.0 GPa

EVA: Ethylene-vinyl acetate; SBS: Styrene-butadien styrene; PC: Polymeric Component (Hard Insert)

### Parametric Study on Mouthguard’s Design Variables in FE Study

The model of the mouthguard was created by overlaying thin layers (0.5 mm each) on the frontal teeth (from left to right canines) according to their labial surfaces to have a customized fit. The mouthguard model was not extended to palatal and occlusal sides to reduce the complexity of the model as they reportedly have minimal effect on frontal teeth protection during a frontal impact [21, 25]. The mouthguard layers and ice hockey puck were meshed by structured hexahedral elements (aspect ratio < 2). Partial tied contacts [23] were applied between the mouthguard and the canines to mimic customized mouthguard retention and to fix it in front of the teeth during the impact simulations. An automatic surface-to-surface contact was defined for the mouthguard-to-teeth interface in the remaining segments, including the labial side of incisors and lateral incisors. The soft component of mouthguards was modelled by Simplified Rubber formulation (MAT181) [26] with characterized loading-unloading curves in compression and tension. Hard insert was modelled either as a simple elastic material or a plastic material with different behaviours in compression and tension (MAT124) [26]. We then evaluated the role of different design variables of a mouthguard such as thickness, material properties and structural design on its protective performance by changing the respective parameters in the model. To find the best mouthguard design, we analysed and compared the transferred stress to teeth protected with different mouthguard configurations. The total thickness of the mouthguard was set either to 3–4 mm, and the hard and soft layers were adjusted accordingly (i.e., 1 mm for the hard insert and 3 mm for the soft component). The stiffness of the soft and hard layers was also modulated in different simulations. To study the effect of space inclusion, respective elements of the standard mouthguard model in front of incisors were removed to generate a 1 mm space. We have also assessed the effect of hard and soft layers attachment (permanent vs. removable) on mouthguard protectiveness. To mimic permanently bonded conditions, a tied contact was defined between the soft component and the hard insert at their interfaces. In contrast, surface-to-surface contact was defined at interfaces to estimate respective contact forces when the hard insert was fitted in the soft component by interlocking features.

### Fabrication of Customized Mouthguards for Experimental Impact Tests

Single-material and multi-material mouthguards were fabricated by conventional thermoforming or by DLP (digital light processing) 3D-printing methods. In particular, DLP is a relatively fast 3D-printing process that projects a patterned light source according to an image of a digital model in the XY plane on the build platform at each Z layer to form a 3D shape. Miicraft® Prime 150y 3D-printer was employed for mouthguard fabrication using biocompatible resins with 100 μm layer thickness precision. The single-layer mouthguards and the soft components of the multi-layered mouthguards with and without space inclusion were 3D-printed using the biocompatible KeyOrtho-IBT (Keyprint®) resin. The hard inserts for the impact protective region in the multi-layered mouthguards were 3D printed using the biocompatible ST1400 (BASF®) resin. The hard and soft components were interlocked together, thanks to the considered protrusions and grooves in their printing model, to form a multi-material mouthguard. In parallel, an experienced dentistry technician prepared the conventional customized mouthguards (according to Erkodent® instructions for Playsafe Heavypro mouthguards) by thermoforming 2 mm and 4 mm EVA sheets sandwiching 0.8 mm SBS film in between.

### Experimental Impact Tests on Fabricated Mouthguards

**Fig. 2:**
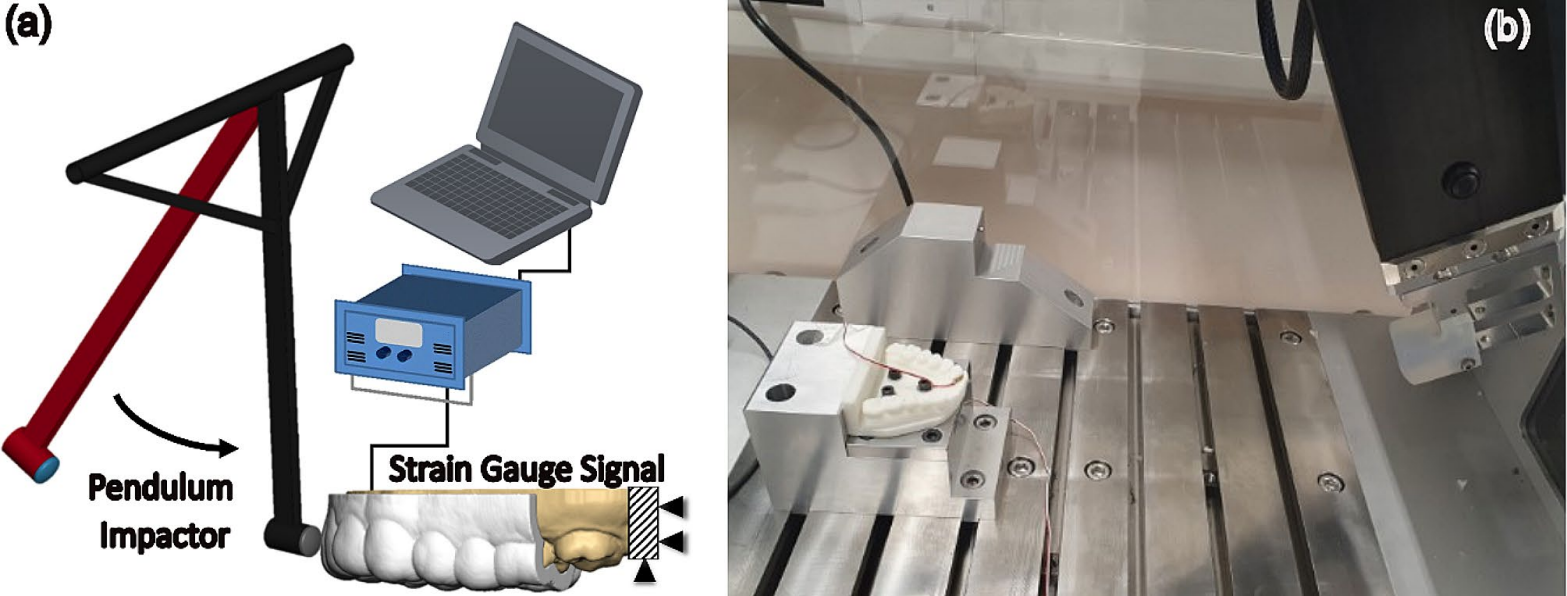
Schematics of impact test scenario on a mouthguard (**a**) and developed impact test setup (**b**)

A pendulum-like test rig was developed (with a 50 cm arm length) using a standard Impetus testing device (4a engineering-Austria). The pendulum head (120 gram) was designed to receive exchangeable impactor materials and/or geometries. An impactor (40 gram) with similar impact-side geometry and hardness (Shore 40D) to an ice hockey puck was 3D-printed with a flexible GR18.1 resin (printodent®) and mounted on the pendulum head as shown in Fig. 2. A solid dental model (E = 2.6 GPa) was 3D-printed with a White-v4 resin (formlabs®) and fixed to the Impetus device test bed using a rigid support for impact tests. The back side/palatal surface of the right incisor in the dental model was equipped with a miniature strain gauge presenting an internal electrical resistance of 350 Ω and 2.54 × 3.19 mm² grid size (Micro-Measurements, North Carolina) using 1-Z70 adhesive (HBM, Germany). The strain gauge was oriented parallel to the tooth-long axis and integrated into a quarter-bridge Wheatstone circuit. To precisely balance the bridge and to compensate for the environmental temperature effect, three dummy strain gauges (not subjected to impact) with identical resistance were used to complete the Wheatstone bridge’s legs. The sensor signal was amplified (DC-92D, Tokyo Sokki Kenkyajo, Japan) and digitized by an analog to digital convertor (NI 9215, National Instrument, Texas) at a 10 kHz sampling rate. A custom LabView code was developed to monitor and record the digitized impact signals. The protective performance of the mouthguards was determined by calculating the ratio of the strain gauge peak voltage with and without the mouthguard. We used the bare dental model strain as the reference value and calculated the ratio of the measured strains with respect to that reference. At least 3 impacts were analyzed for each tested configuration. We fabricated two mouthguards for each group. The impact response of the tested mouthguards were repeatable and we could not see any effect of ageing on protectiveness of mouthguard even after 5 impacts in our tested configuration. The relative strain values were statistically analyzed by t-test between groups (p-value = 0.05, *n* = 3–5).

## Results

### Impact Simulation and Role of Mouthguard Design in Teeth Protection

**Fig. 3:**
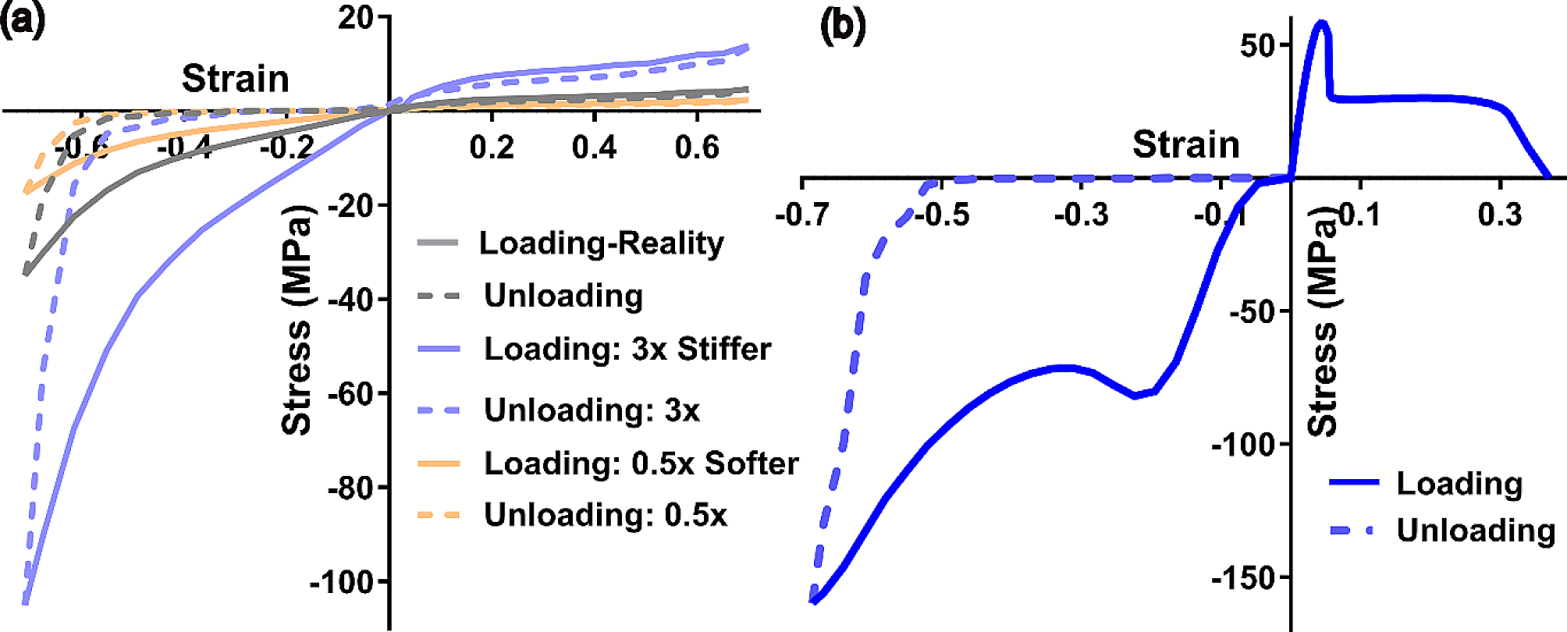
Representative compression and tension stress-strain curves for the soft EVA (**a**) and hard SBS (**b**) samples (EVA: Ethylene-vinyl acetate; SBS: Styrene-butadien styrene). The stress values of EVA curves were scaled up and down to represent stiffer and softer behavior for the rubber-like component of a mouthguard in simulations

Following a mesh convergence study, hourglass energy control, and energy balance verification, we examined the protective performance of different mouthguards subjected to a puck impact. For this purpose, we directly characterized the mechanical properties of the soft and hard materials used for the conventional Playsafe mouthguards fabrication and employed them in our finite element model. Figure 3 illustrates the stress-strain curves of the Ethylen-Vinyl-Acetat (EVA), and Styrene-Butadien-Styrene (SBS) samples extracted from Erkodent® sheets. While the rubber-like EVA sample was tear-resistant and sustained large elongation, the hard SBS sample entered under plastic deformation in compression and failed under tension after 20% of strain. For simulation purposes, we also numerically scaled up (3x) and down (0.5x) the measured stress values for EVA material to study the effect of stiffer or softer rubber-like components on the mouthguard performance. The SBS hard layer was also replaced with a stiffer polymeric component (i.e., E = 2.4 or 3.0 GPa) to evaluate the effect of the hard insert’s degree of hardness on mouthguard performance.

**Fig. 4:**
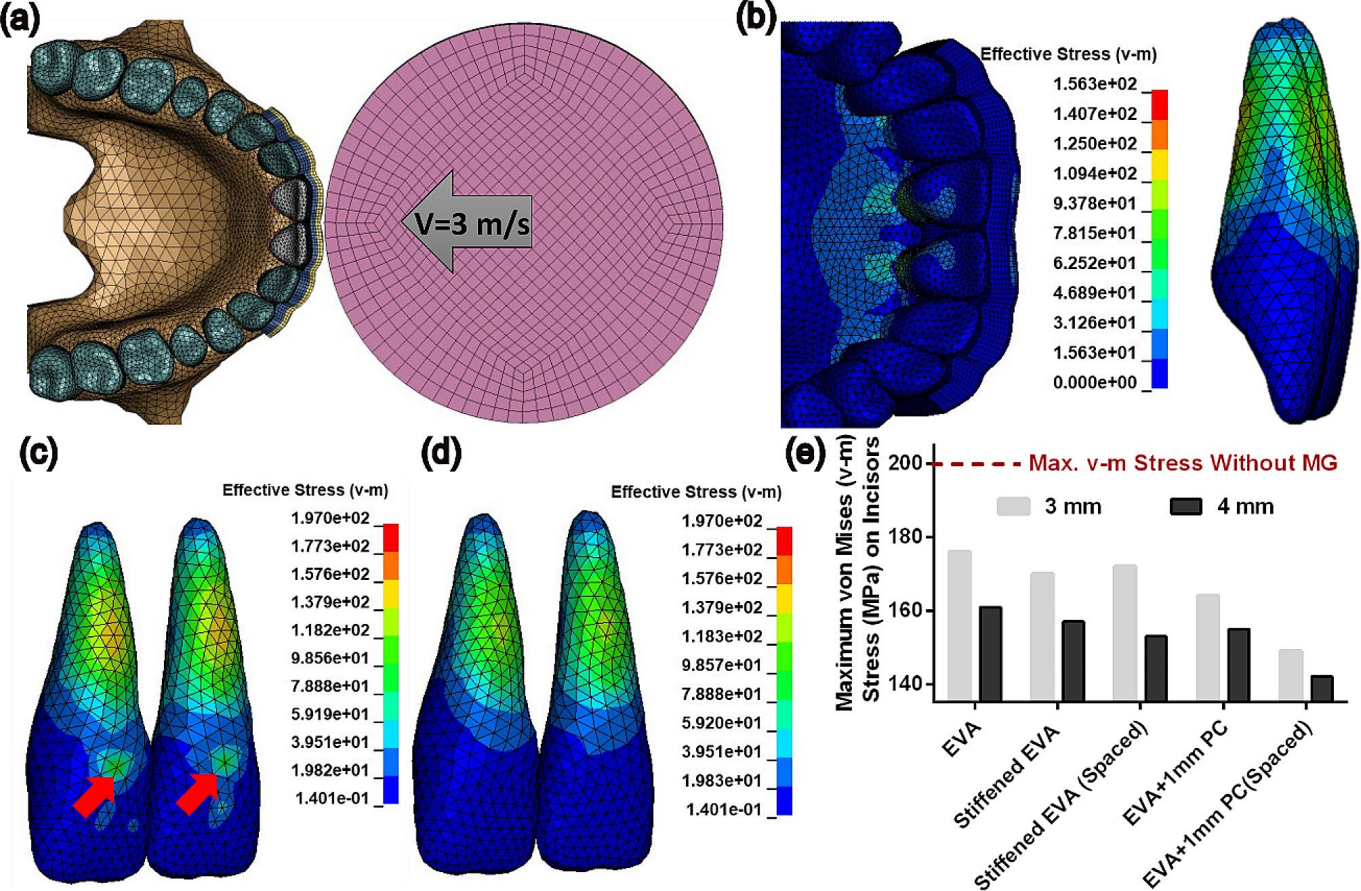
(**a**) Dynamic finite element simulation of puck impact to teeth protected with a representative 3 mm mouthguard composed of a soft layer (blue), hard insert (yellow), and space in front of the incisors. (**b**) Representative visualization of the transferred von Mises (v-m) stress to teeth protected with a standard 4 mm mouthguard composed of single EVA material. Maximum effective stress field on incisors during puck impact without (**c**) and with (**d**) mouthguard protection showing higher transferred stress to crown and root when a mouthguard is not present (red arrows). (**e**) Comparison of maximum von Mises stress in incisors following impact with 3 mm and 4 mm mouthguard designs (EVA: Ethylene-vinyl acetate; PC: Polymeric Component-Hard Insert)

Our simulation results revealed that the incisors’ roots sustain the maximum mechanical stress during a frontal impact. Regardless of the mouthguard’s presence or design, the impact load generated tensile stress on the anterior side of the root and compressive stress on the posterior side. Representative von Mises stress fields on incisors at the time of peak stress are illustrated in Fig. 4, with and without mouthguard protection. While the effective stress on the incisor crown at the puck impact locations is significant without mouthguard protection (shown by arrows in Fig. 4-c), the stress on the incisor crown is minimal with mouthguard protection (Fig. 4-d). By comparing the transferred stress to the teeth in different mouthguard designs, we consistently observed that the larger mouthguard thickness is more efficient in reducing the risk of injury in all designs with similar material and structural configurations (Fig. 4-e). However, our simulations showed that higher protection could be obtained in thinner designs than thicker ones if we modify the mouthguard design (e.g., EVA vs. EVA + 1 mm PC (Spaced) in Fig. 4-e).

**Fig. 5:**
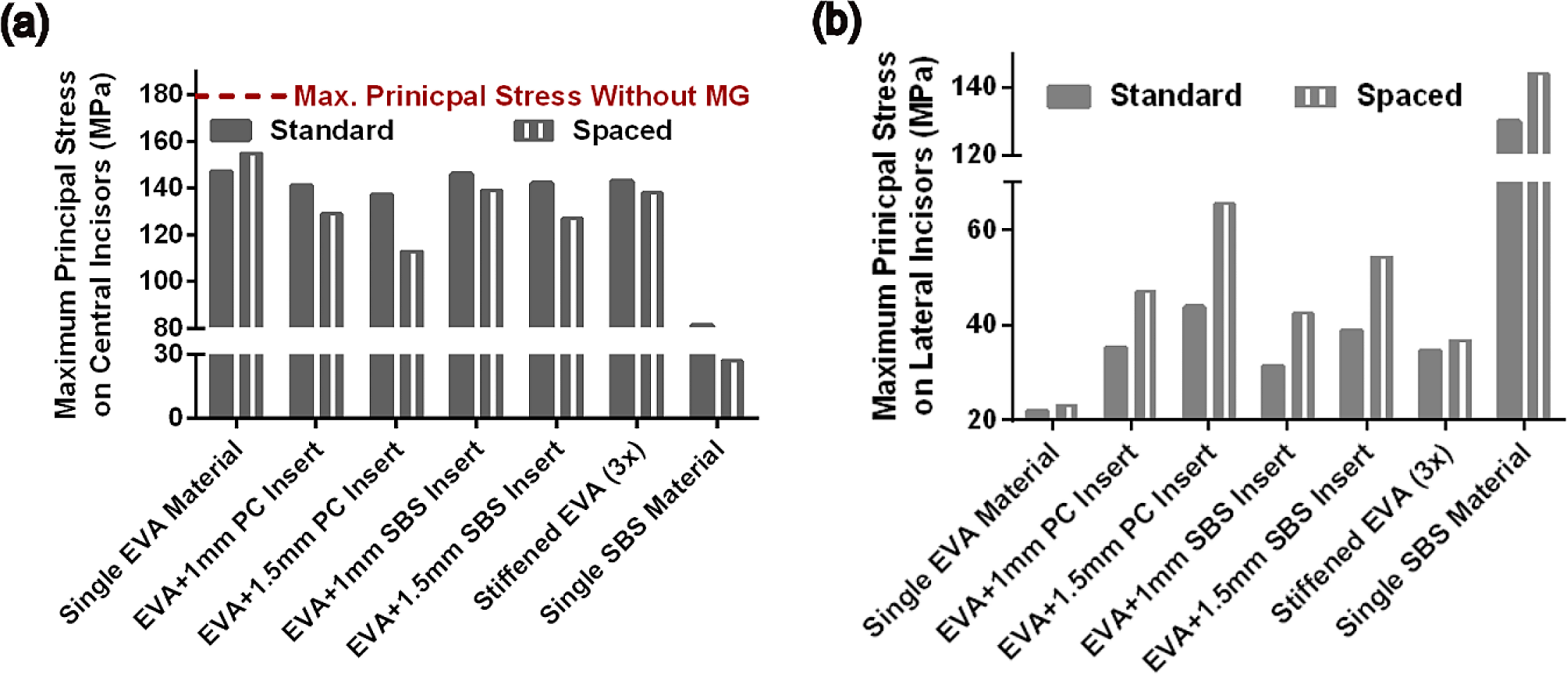
Effect of space inclusion (1 mm) as well as hard insert modulus (PC: E = 2.40 GPa vs. SBS: E = 1.8 GPa) and thickness (1 mm vs. 1.5 mm) on incisors and lateral incisors protection in different mouthguard designs with 4 mm thickness (EVA: Ethylene-vinyl acetate; SBS: Styrene-butadien styrene; PC: Polymeric Component). Results show that higher protection is achieved in spaced mouthguard designs consisting of a harder insert in the frontal region

We found that the addition of a hard insert to the soft layers could not noticeably change the protective performance of the mouthguard when the teeth are in full contact with the mouthguard (standard design). In this case, the effect of a hard insert can be slightly detrimental or beneficial depending on the stiffness and thickness of the hard insert. For example, a 1 mm SBS layer (E = 1.8 GPa) in front of a 3 mm EVA layer could not reduce the maximum principal stress on the incisors (Fig. 5-a) compared to the single 4 mm EVA mouthguard (147 MPa). However, inserting a harder and thicker polymeric component (1.5 mm PC insert: E = 2.4 GPa) can slightly reduce the maximum stress on the incisors from 147 to 137 MPa. The best protection was observed when we employed hard and soft layers in combination with a 1 mm space in front of the incisors (spaced design). In this configuration, the protective performance of the mouthguard could be tuned, by controlling the hard layer’s geometry and elastic modulus. Our simulation showed that the maximum principal stress on incisors was significantly reduced from 180 MPa (without mouthguard protection) to 113 MPa by using a 4 mm mouthguard composed of 1.5 mm PC insert (E = 2.4 GPa) and a 1 mm space in front of the incisors (shown by arrow in Fig. 5-a). Moreover, our results showed that a hard material can better contribute to impact load distribution on teeth than a soft material (Fig. 5-b). For instance, the maximum stress in the lateral incisor is significantly higher in the design consisting of a 1.5 mm PC hard insert compared to a single material EVA mouthguard. However, when there is only a single hard material, the stress on lateral incisors is drastically increased indicating that they sustain the dominant portion of the impact load. This implies that the protective performance of a mouthguard fabricated with a single hard material is not optimal.

**Fig. 6:**
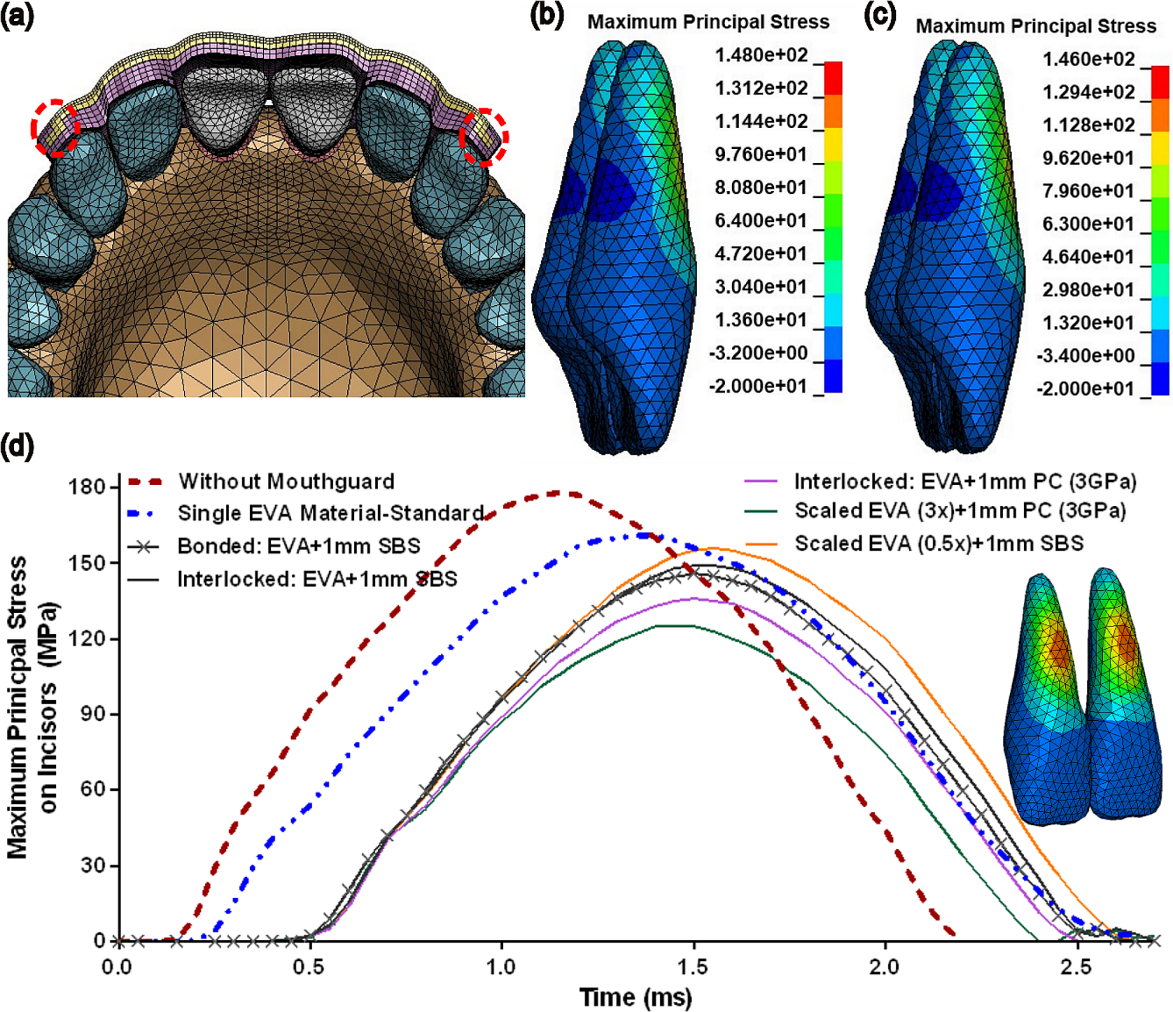
Finite Element simulation and analysis of the protective performance of multi-layered mouthguards with a space inclusion (EVA: Ethylene-vinyl acetate; SBS: Styrene-butadien styrene; PC: Polymeric Component). (**a**) Design configuration with interlocking features for hard (yellow) and soft (purple) layers defined by a surface-to-surface contact algorithm on the interface of removable components. Qualitative evaluation of impact-induced stress fields on incisors protected with multi-layered mouthguards consisting of permanently bonded (**b**) or interlocked (**c**) components. (**d**) Evolution of impact-induced maximum principal stress on central incisors with and without mouthguard protection. The thickness of all mouthguards was fixed at 3 mm. The presence of a standard mouthguard (without space in front of incisors) composed of a soft EVA material enlarged the time of impact (dash double-dotted blue line) compared to the unprotected impact condition (dashed red line). The space inclusion delays the time of stress evolution on incisors following the impact onset (all solid lines). Examination of different configurations shows that the mechanical properties of each component contribute to the protective performance of a mouthguard with removable parts (e.g., solid orange, purple, and green lines)

We also evaluated the role of linkage between soft and hard components in the protective performance of multi-material mouthguards. For this purpose, we evaluated the impact response of teeth protected with 3 mm mouthguards consisting of permanently bonded layers (Fig. 4-a) or mechanically interlocked components (Fig. 6-a). While the hard and soft layers were bonded with tied contact definitions in the permanently bonded layers, automatic surface-to-surface contact definitions were employed between removable components. Importantly, we observed a comparable stress field on impacted teeth in the permanently bonded (Fig. 6-b) and interlocked (Fig. 6-c) configurations. By analyzing the maximum principal stress evolution over the impact time, we confirmed a similar protection level in the two linkage designs (Fig. 6-d, solid black lines with and without cross symbol). We also verified that the protective performance of a mouthguard with removable components can be controlled by the mechanical properties of each component. For example, higher protection was obtained when we used a stiffer polymeric component than SBS (E = 3.0 GPa vs. E = 1.8 GPa) as a hard insert (Fig. 6-d, solid purple and blue lines). Similarly, when the stiffness of the soft component is scaled up or down (e.g., scaled EVA:3x or 0.5x), higher or lower protections were observed, respectively (Fig. 6-d, solid orange and green lines).

According to these observations, one can conclude that the hard insert initially distributes the impact load. The space inclusion postpones the time of stress evolution on incisors and increases the contribution of other teeth to sustain the impact load. Additionally, impact energy is partially absorbed by bending of the frontal region components when there is a space between the frontal teeth and the mouthguard. In parallel, the impact load is absorbed by the soft layer’s compression, and the time of impact is extended.

### Experimental Impact Tests

**Fig. 7:**
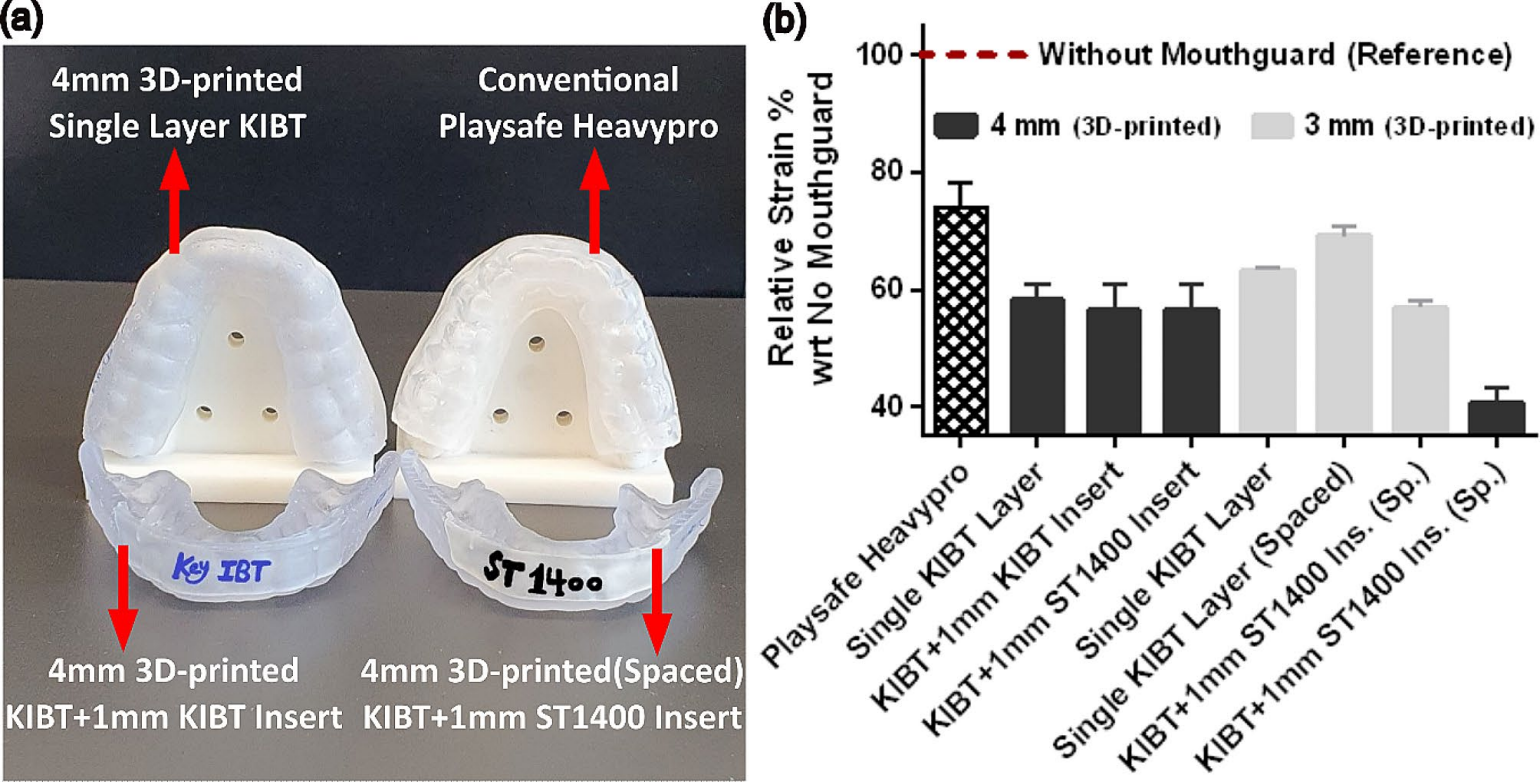
(**a**) Representative 3D-printed and conventional Heavypro customized mouthguards. (**b**) Experimental results with an impactor speed of 3 m/s show that 3D-printed mouthguards with a hard insert and space in front of the incisors provide higher protection among different design configurations, including Playsafe Heavypro® (KIBT: KeyOrtho IBT resin; Ins: Insert; Sp.: Spaced; wrt: with respect to)

To confirm our simulation findings, we performed experimental impact tests on a 3D-printed dental model protected with different mouthguards. It is important to note that we cannot directly measure the stress on human teeth through an impact test as we did in the simulations. However, we can compare the transmitted strain on the impacted incisors with different mouthguard configurations and analyze their protectiveness. Accordingly, we performed tests with a puck-like impactor at a speed of 3 m/s on different design configurations. Representative 3D-printed Key-OrthoIBT mouthguards and corresponding relative strains following the impact tests are shown in Fig. 7. The obtained results from the impact tests were consistent with our simulation observations. Among different tested configurations, maximum protection is achieved with the design presenting a hard insert in front and an intentional space between the incisors and the soft component of the mouthguard.

Similar to our simulation results, the hard insert addition could not remarkably improve the protective performance of the mouthguard compared to the single-layer design. Specifically, the relative strain on the incisors was reduced by around 40% in the single layer 4 mm mouthguard 3D-printed with KeyOrtho IBT resin and when a 1 mm hard insert (ST1400: E = 1.9 GPa) was added without space inclusion (*KIBT + 1 mm ST1400 insert*). In parallel, the space inclusion in front of the incisors slightly reduced the protective performance of the 3 mm mouthguard 3D-printed with KeyOrtho IBT resin. However, when the space inclusion was combined with the hard layer addition in the 3 mm mouthguard designated by *KIBT + 1 mm ST1400 ins. (Sp.)*, its protective performance was significantly improved. This implies that the space inclusion could be beneficial or detrimental depending on the mouthguard stiffness in the frontal region. Notably, the relative strain on the incisors was reduced by more than 55% when protected with the spaced type of a 4 mm mouthguard composed of soft KeyOrtho IBT and hard ST1400 layers.

Our impact tests revealed that the conventional Playsafe Heavypro mouthguard is less protective than the 3D-printed mouthguards and it could only attenuate the transmitted strain by around 25%. Surprisingly, even the single layer 3 mm mouthguard 3D-printed with KeyOrtho IBT resin performed better than the Playsafe Heavypro mouthguard fabricated by lamination of two EVA and one SBS sheets. This can be explained by the fact that the covalently crosslinked KeyOrtho IBT material is stiffer than EVA thermoplastics. Consistent with our simulations, our impact tests confirmed that the larger mouthguard thickness is more protective in the designs with similar material and structural configurations. For example, the transmitted relative strain is higher in the 3 mm single-layer mouthguards than in its 4 mm version. This is also true when we compare the performance of 3D-printed mouthguards consisting of a 1 mm hard insert and space in their structure. In contrast, the spaced 3 mm mouthguard composed of KeyOrtho IBT and ST1400 resins marginally performed better than the 4 mm single-layer mouthguard 3D-printed with KeyOrtho IBT resin. Last, we measured comparable strain on the impacted incisor with the 4 mm mouthguards 3D-printed by KeyOrtho IBT resin in single-layer or double-layered (KIBT + 1 mm KIBT Insert) forms. Yet, the former was 3D-printed uniformly and the latter was created with separately printed components that were mechanically interlocked together. These measurements validate our simulations and demonstrate that similar protective performance is obtained in the designs with permanently bonded or removable components. Accordingly, the choice of the materials is no anymore limited to chemically bondable compositions, providing more freedom from the material selection perspective.

## Discussion

Literature suggests that dental injuries are prevalent worldwide in different sporting activities including American football, ice hockey, rugby, judo, basketball, handball, and soccer with an incidence rate from 9 to 45% [1]. For example, the dental injury risk is estimated at around 13% for soccer players in Japan [27], and 33% for ice hockey players in Switzerland [28]. Similarly, it is reported that a significant portion of dental injuries (39%) occur during sport-related accidents in the US [29]. These statistics rationalize the recommendation of the American Dental Association for mouthguard wearing in 29 sports [30] to reduce the risk of associated dental injuries. Despite the availability of three types of mouthguards (e.g., off-the-shelf, mouth-formed, and custom-made), literature findings suggest that only well-designed customized mouthguards can simultaneously provide efficient protection and comfort [3, 4, 31]. To obtain an insight into the contributing factors to the protectiveness of customized mouthguards, we compared the performance of different configurations utilizing numerical and experimental evaluations. Our simulation findings were in close concordance with the corresponding experimental evaluations. It was demonstrated that the 3D-printed mouthguards could offer higher protective capabilities than conventionally fabricated mouthguards. Our finite element model enabled us to design more efficient mouthguards that can be created with 3D printing to get the desired performance. Over the last few years, 3D printing techniques have increased the possibilities for accurate fabrication of customized mouthguards with promising benefits [4, 6, 9, 32]. Among all tested 3D-printable materials, flexible Key OrthoIBT and rigid ST1400 resins showed the best results for our design realization thanks to their superior mechanical properties (e.g., tear and impact resistance capability) and biocompatibility. A recent study also found Key OrthoIBT to be the most suitable photocurable resin for the mouthguard application after comparing the properties of different flexible resins [5].

The ideal mouthguard should minimize the transferred stress to teeth following a direct impact. In parallel, the device’s thickness must be kept as thin as possible for the users’ comfort. We learned from our simulations that a harder frontal region could better distribute the load and absorb impact energy through bending if we include a space between the frontal teeth and the mouthguard. In parallel, we observed that a softer layer could extend the impact time and absorb its energy by compression which was also reported in previous studies [20]. This in-silico step could shed light on the optimal mouthguard design for effective load distribution and shock absorption. Thanks to the results of our numerical model, we demonstrated that a better performance might be achieved in thinner configurations if we optimally design the structural and material properties of the mouthguards. For example, the thinner 3 mm 3D-printed mouthguard with a 1 mm hard insert and a space in its structure performed better than bulkier multi-layered Playsafe Heavypro mouthguards (non-uniform larger thickness ≥ 4 mm). However, it remains to be determined to what extent this configuration is viable when subjected to different impact objects or energies. Indeed, depending on the impact conditions, the relative contribution of the mouthguard to protect teeth could be different as its capacity to sustain and attenuate the impact load is not boundless.

In agreement with previous experimental and numerical studies [11–14], we also observed that the larger the mouthguard thickness is, the more efficient it is in reducing the risk of injury in a specific material and structural configuration. Importantly, our developed finite element model for impact simulations and 3D-printing-based fabrication allowed us to compare the protective performance of different structural and material configurations at determined thicknesses. Indeed, with the conventional thermoforming method, it is not possible to assess the effects of isolated space and/or hard insert incorporation without changing the total thickness of the customized mouthguard [11, 19]. Our numerical and experimental evaluations indicated that isolated space inclusion or hard insert incorporation is not necessarily beneficial in all design configurations. However, their combination could significantly modulate the protective performances of the mouthguards in a synergetic manner.

Higher energy impacts might occur during different sporting activities [11] than what we simulated or tested. However, the system’s boundary conditions in real-world impacts differ from what we can reproduce in a laboratory setup. For example, jaw and neck compliance and head motion can significantly reduce the transferred shock compared to the fixed boundary condition that we applied to the dental model. Moreover, the support of lower arch teeth and available soft tissues such as lips, PDL, and gingiva can attenuate the load on the affected tooth [11, 20, 33]. Our numerical models can, therefore, only partially reproduce the real case situations. We are also aware of the limitations of the employed rigid dental model in our impact tests to represent the actual parameters of a human teeth-jaw system. Nevertheless, our primary goal was to compare the performances of different mouthguards in similar impact conditions. Our results allowed us to estimate and discriminate the protective capacities of the tested mouthguards. We intentionally employed the impacted incisor strain values to compare different configurations’ performance. Previous studies showed that transmitted strain better discriminates the shock absorbency of different mouthguards and materials thickness than force or acceleration sensors [16, 18, 20]. Moreover, the literature suggests that similar and consistent results could be obtained to compare and rank mouthguards’ protective performance regardless of the strain sensor position (Palatal or Labial side of incisor) [18, 34].

## Conclusion

In summary, combining finite element simulations, additive manufacturing, and impact tests is valuable for developing functional mouthguards with higher protective performances and user comfort. The proposed 3D-printed mouthguard combining a spaced rail made of Key IBT resin and an insert made of ST1400 resin showed high capability for teeth protection and performed much better than conventional customized Heavypro mouthguards, even with a thinner thickness. This specific design was optimal for distributing the impact load and absorbing the impact energy through hard insert bending and soft layer compression. We envision the future with printable composite custom-mouthguard presenting distinct attributes in different regions that are adaptable by the user based on the level/type of competition and associated harshness of the impact incidences.

## Data Availability

Simulation and experimental data will be made available on request.

## Abbreviations

EVA: Ethylene-vinyl acetate
SBS: Styrene-butadien styrene
MG: Mouthguard
FE: Finite element
PC: Polymeric Component
Ins.: Insert
PDL: Periodontal ligament
v-m: Von mises
Sp.: Spaced
KIBT: KeyOrtho IBT resin
ST: ST1400 resin
Wrt: With respect to
